# Daily Emotional Well-Being and Cardiometabolic Syndrome in Adulthood: The Role of Sleep Deficiency

**DOI:** 10.1093/geroni/igaa057.2176

**Published:** 2020-12-16

**Authors:** Hye Won Chai, Susanna Joo, Sun Ah Lee, David Almeida

**Affiliations:** 1 The Pennsylvania State University, University Park, Pennsylvania, United States; 2 Yonsei University, Seoul, Republic of Korea

## Abstract

Previous studies note daily emotional well-being and sleep duration as significant correlates of cardiovascular health including cardiometabolic syndrome. However, not much is known about the interactive effects of emotional well-being and sleep. Expanding upon current research, this study examined whether sleep deficiency, defined as having on average <7 hours of sleep a day, moderated the associations between daily emotional well-being and cardiometabolic syndrome. Data was drawn from the Midlife in the United States Biomarker Project and the National Study of Daily Experiences (N = 1,163; Mean age = 53.3). Results showed significant interaction effects- higher negative affect was associated with worse cardiometabolic syndrome and higher positive affect was associated with better cardiometabolic syndrome only among those with deficient sleep. Such association was stronger for older adults compared to young adults. These findings suggest that individuals with insufficient sleep are more affected by health-related benefits and costs of daily emotional experiences.

